# Uncovering Insights Into the Biology of *Mycobacterium tuberculosis* Using Genetic Tools

**DOI:** 10.1002/mbo3.70206

**Published:** 2025-12-21

**Authors:** Alessandro Stamilla, Deborah Recchia, Giovanni Stelitano, Ludovica Maci, Maria Concetta Marturano, Edda De Rossi, Laurent Roberto Chiarelli, Maria Rosalia Pasca, Giulia Degiacomi

**Affiliations:** ^1^ Dipartimento di Biologia e Biotecnologie “Lazzaro Spallanzani” Università di Pavia Pavia Italy; ^2^ Fondazione IRCCS Policlinico San Matteo Pavia Italy

**Keywords:** CRISPR‐Cas system, gene knockdown techniques, gene targeting, genetic engineering, *Mycobacterium tuberculosis*, recombination

## Abstract

*Mycobacterium tuberculosis* (*Mtb*), the etiological agent of tuberculosis, is one of the most challenging pathogens due to its complex physiology, diverse clinical manifestations, and growing multidrug resistance. The global rise of drug‐resistant *Mtb* strains has prompted the search for innovative genetic and molecular strategies to accelerate drug discovery and vaccine development. Progress in *Mtb* research has long been hindered by its slow replication rate and impermeable cell envelope, which limit the efficacy of genetic manipulation. This review outlines methodological advances that have transformed the study of *Mtb* pathogenesis and drug resistance mechanisms. Traditional homologous recombination–based approaches, including allelic exchange and specialized transduction, laid the groundwork for targeted mutagenesis but were limited by low efficiency. The advent of phage‐derived recombineering systems, such as the Che9c RecET, has substantially improved the precision and throughput of genetic modification. Hybrid systems such as ORBIT, which combines oligonucleotide‐mediated recombineering with Bxb1 integrase, have further enabled rapid and versatile genome engineering across mycobacterial species. Parallel developments in conditional gene expression systems (e.g., the use of TetR/Pip‐based promoters) have facilitated the functional analysis of essential genes and the validation of novel drug targets. The advent of CRISPR–Cas technologies has represented a paradigm shift, by enabling programmable, high‐fidelity gene regulation and functional genomics even in slow‐growing mycobacteria. Together, these genetic innovations are transforming *Mtb* research by accelerating drug discovery and vaccine design, and shedding light on host–pathogen interactions.

## Graphical abstract

Genetic manipulation tools such as homologous recombination, CRISPR‐Cas systems, and gene knock‐out/knock‐down strategies have advanced *Mycobacterium tuberculosis* (*Mtb*) research, despite challenges posed by its slow growth, complex pathology, and drug resistance. The sequencing of the H37Rv genome enabled molecular approaches that revolutionized the field, providing key insights into *Mtb* biology, pathogenesis, and drug action. These innovations continue to support the development of new drug therapies and a deeper understanding of host‐pathogen interactions in TB.

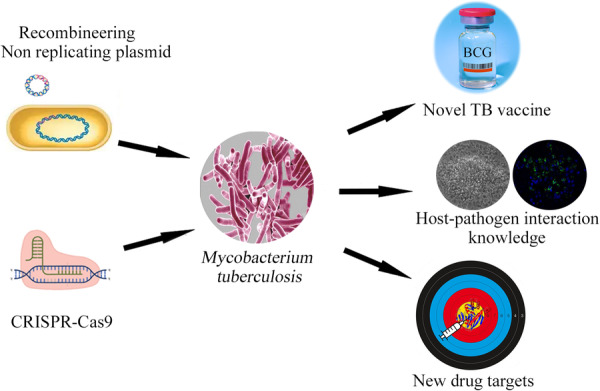

## 1 Introduction

Tuberculosis (TB), caused by *Mycobacterium tuberculosis* (*Mtb*), poses many challenges in its treatment due to various clinical manifestations (latent TB, pulmonary TB, extra‐pulmonary TB, etc.) and growing drug resistance. In the last decade, the global TB incidence declined by only ~8.3% reaching 10.8 million cases in 2023. Meanwhile, TB mortality dropped by ~23% over the same period, with ~1.25 million deaths estimated in 2023. The significant challenge of TB control is highlighted by these modest gains. Fortunately, this decline in the overall number of deaths caused by TB in 2023 consolidates the positive trend found in 2022, following the significant increases during the COVID‐19 pandemic (2020–2021). Current therapy for drug‐sensitive TB includes a 6‐month regimen of four drugs; however, drug‐resistant *Mtb* isolates are difficult to treat, requiring a complex and prolonged treatment, hindering progress in containing the global TB epidemic (World Health Organization 2024). Multidrug‐resistant (MDR‐TB) and extensively drug‐resistant *Mtb* strains are indeed becoming increasingly prevalent. Treatment remains lengthy, toxic, and often less effective, sometimes requiring 18–24 months of therapy. In response, the scientific community has been trying to develop new drug regimens that are safer, faster, shorter, and more effective against both drug‐sensitive and drug‐resistant *Mtb* isolates. Without the discovery of novel drugs and innovative approaches for drug development, the progress made in TB control could be reversed. First‐line regimens (isoniazid, rifampicin, pyrazinamide, ethambutol) are over 40 years old and require at least 6 months. This prolonged duration often leads to poor patient adherence and disease relapses. Most recently, new recommendations have been implemented for the treatment of drug‐sensitive TB, involving a new 4‐month treatment regimen (isoniazid, rifapentine, moxifloxacin, pyrazinamide) (Saukkonen et al. 2025). However, the increased burden of daily pills and existing fluoroquinolone resistance are potential issues (Wilson et al. 2025). Consequently, developing shorter, safer, and more potent regimens is crucial, which depends on identifying new drug targets and improving our understanding of *Mtb* physiology. Advances in genetic and molecular tools now enable researchers to dissect the complex biology of this pathogen, identify genes essential for bacterial survival, elucidate drug mechanisms of action and resistance, and validate novel therapeutic targets to support rational drug design. Owing to the sequencing of the *Mtb* H37Rv genome (Cole et al. 1998), traditional methodologies in mycobacteriology have been progressively replaced by molecular techniques. These advances have paved the way for new approaches to studying *Mtb* biology, particularly its pathogenesis and the mechanisms of action of antitubercular drugs. However, the thick, waxy, and hydrophobic *Mtb* cell wall poses a barrier to DNA uptake, limiting its genetic manipulation. The impermeable nature of the mycobacterial cell envelope poses multiple difficulties for drug discovery efforts. These include: screening limitations related to the impossibility of crossing the cell wall to reach their intracellular targets; target validation issues linked to the genetic tools that rely on efficient DNA uptake or recombination being hampered by the same barrier, complicating genetic manipulation and functional studies; pharmacokinetic constraints whereby drugs that do reach the bacillus often exhibit poor diffusion and accumulation. This necessitates higher doses or prolonged treatment to maintain therapeutic efficacy (Chauhan et al. 2024).

Furthermore, the complexity and time required for *Mtb* genetic manipulations have frequently hindered the implementation of such tools (Lamrabet and Drancourt 2012).

Despite these challenges, genetic approaches such as knock‐down and knock‐out strategies are actively being used by researchers both to accelerate the identification and characterization of potential drug/vaccine targets and to obtain a deeper knowledge of how *Mtb* establishes an infection and causes disease.

This review aims to explore the genetic tools used to investigate the *Mtb* pathogenesis as well as the mechanisms of action and resistance of antitubercular drugs, with a particular focus on the most recent and innovative strategies.

## 2 Gene Replacement

Several methods for targeted gene disruption in the *Mtb* genome have been developed in recent years; many strategies are based on homologous recombination (HR), while other approaches were developed to be recombination‐independent (i.e., CRISPR/Cas).

**Figure 1:**
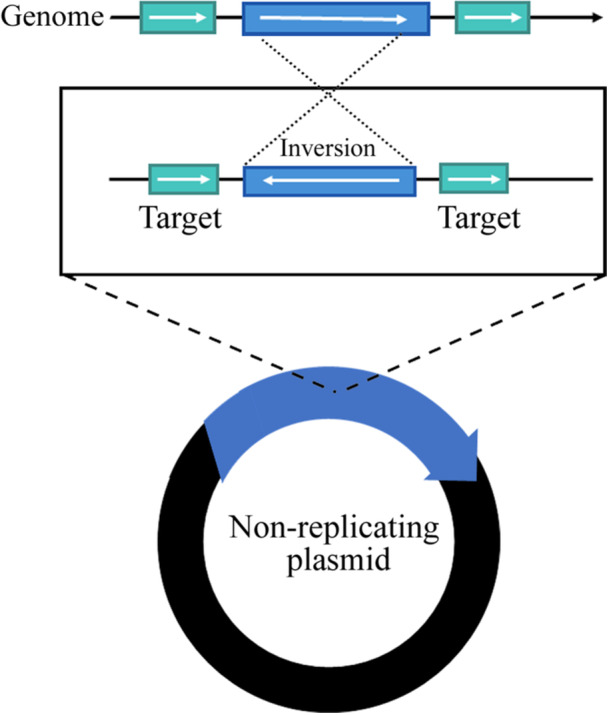
Construction of knock‐out mutant using a non‐replicating plasmid. A non‐replicating plasmid delivers a homologous DNA sequence (blue) that replaces the target gene through double cross‐over recombination between two target sequences (green), resulting in a knock‐out mutant.

Among the three DNA repair pathways in mycobacteria, HR is chiefly mediated by the RecA‐dependent pathway, which facilitates targeted gene replacement via strand exchange between homologous regions. Over 30 years ago, the first successful attempts to remove a gene from the *Mtb* genome were achieved by taking advantage of HR (Aldovini et al. 1993; Pelicic et al. 1997). HR is performed by a dedicated enzymatic system that catalyses the exchange of two similar DNA sequences (Gupta et al. 2017). This process has been widely adopted to generate gene knockouts by allelic exchange, facilitated by the use of selectable and subsequently counterselectable markers. Basically, a targeted region in the genome is replaced with a homologous DNA sequence that can be delivered by a non‐replicating plasmid. In this approach, this plasmid harbors a selectable marker gene flanking regions of the target gene. Once transformed into the mycobacterial cells, this undergoes double cross‐over recombination events, replacing the wild‐type gene with a modified sequence, resulting in a knock‐out mutant (Figure 1) (Hinds et al. 1999; Parish et al. 1999).

The allelic replacement method is a genetic technique used to specifically modify a gene or a genomic region by replacing an existing allele with a modified one. This method is particularly useful for studying the function of a gene in a pathogenic microorganism such as *Mtb*, as it allows specific mutations to be obtained without inserting foreign sequences, which could alter gene expression in an uncontrolled manner. One of the first examples of a metabolic mutant obtained by allelic exchange was a strain in which *icl1* was deleted. *icl1* encodes the glyoxylate shunt enzyme isocitrate lyase essential for metabolism of even chain fatty acids and it was reported to exhibit an in vivo survival defect (McKinney et al. 2000). Starting from here, subsequent studies confirmed an essential role for isocitrate lyase showing that the lipid metabolism is important for the growth and survival of *Mtb* during all phases of its life cycle (Ehrt et al. 2015).

This technique has then evolved to generate untagged mutants, addressing various research and clinical needs. These include the development of potential vaccine strains, avoiding a polar effect on the expression of downstream genes in an operon, or enabling the re‐use of a specific marker for further genetic manipulation (Malaga et al. 2003).

Among the methods developed to generate unmarked mutants (Pavelka and Jacobs 1999; Parish and Stoker 2000; Bardarov et al. 2002; Malaga et al. 2003), the two‐step pNIL/pGOAL system is one of the most recognized and utilized (Parish and Stoker 2000). This system employs two plasmid series: the pGOAL vectors, which carry selection marker cassettes, such as *lacZ*/*sacB* and a drug‐resistance gene, and the pNIL plasmids, which are used to modify the target gene. Following transformation, colonies harboring the pNIL vector inserted into the genome through the first cross‐over event are selected. To obtain the desired untagged mutant by negative selection, the second crossover event must be selected. This approach not only yields clean, untagged mutants but also facilitates successive rounds of genetic manipulation, including multiple gene deletions.

This procedure has been successfully used by TB researchers, yielding interesting results. For example, an *Mtb* strain mutated in *hspX* gene, coding for the Heat Shock Protein X induced during hypoxia (∼80‐fold; *dosR* dependent), was obtained by using the pNIL/pGOAL strategy. This study demonstrated that HspX is essential for both the stress response and survival in host cells, during *Mtb* infection (Hu et al. 2006). A remarkable application of this technique is the development of the MTBVAC strain, the first live attenuated *M. tuberculosis*‐based vaccine, currently in phase III clinical trials (Luabeya et al. 2025). MTBVAC was obtained by two consecutive and independent deletions of two virulence‐associated genes: *phoP* and *fadD26* (Arbues et al. 2013; Malaga et al. 2003; Jackson et al. 2001; Pelicic et al. 1997). The *phoP* gene was deleted because it encodes a transcriptional regulator that controls the expression of over 2% of the *Mtb* genome, including many genes involved in virulence. The second deleted gene, *fadD26*, is in an operon essential for the biosynthesis and export of phthiocerol dimycocerosates, which are the major cell wall lipids associated with *Mtb* virulence. Together, these deletions significantly attenuate the strain while preserving its immunogenicity, making MTBVAC a promising candidate for improved TB vaccination.

However, a major limitation of these early HR‐based allelic exchange techniques was their low transformation efficiency (Hinds et al. 1999). To overcome this issue, attempts were unsuccessfully made using replicating plasmids instead of suicide vectors to increase the persistence of the constructs within the cells and to allow more time for HR to occur (Pashley et al. 2003). In the two‐plasmid incompatibility system, two plasmids sharing the same origin of replication but differing in their genetic content were sequentially introduced into mycobacterial cells. Because plasmids with identical origins cannot stably coexist in the same host (due to replication incompatibility), the system forced the loss of one plasmid over time. The idea was to first establish a replicating plasmid carrying the homologous arms for recombination, and then introduce a second, incompatible plasmid carrying a counter‐selectable marker. This system aimed to create selective pressure that would favor HR by destabilizing the maintenance of one plasmid. Despite its seeming promise, this method was largely unsuccessful in *Mtb*. The frequency of successful allelic exchange events has, in fact, been reduced drastically, due to the intrinsic inefficiency of the native HR system in *Mtb*, the low transformation efficiency, and its slow growth rate (Pashley et al. 2003).

One of the last attempts consisted of the site‐specific recombination method derived from bacteriophages or transposons, obtaining a significant increase of both mutated allele substitution and unmarked knock‐out generation (Bardarov et al. 2002; Malaga et al. 2003; Song and Niederweis 2007; Jain et al. 2014). In this last case, transformation efficiency was undoubtedly improved using specialized transduction compared to traditional electroporation, although phage construction is still cumbersome, and endogenous HR is not so efficient in slow‐growing bacteria, such as *Mtb*. In fact, Bardarov et al. (2002) demonstrated that specialized transduction is a highly efficient and reproducible method for delivering homologous DNA substrates for allelic exchange in both fast‐ and slow‐growing mycobacteria. Using this approach, they generated seven gene deletion mutants in *lysA, nadBC, panC, panCD, leuCD, Rv3291c,* and *Rv0867c* genes as antibiotic‐resistant transductants across three *Mycobacterium bovis* BCG substrains, three *Mtb* strains, and one *Mycobacterium smegmatis* strain. To obtain unmarked deletion mutants, a plasmid encoding the γδ‐resolvase (*tnpR*) was used to excise the antibiotic resistance cassettes. Upon transduction into host mycobacterial cells, recombination frequencies were around 10^−6^. Consequently, these mutants could not be constructed by electroporation with a suicide plasmid, as the highest electroporation efficiency was about 10^−^^5^. So, these results were encouraging in terms of using specialized mycobacteriophages for efficient in vitro gene replacement in mycobacteria, even if the antibiotic resistance cassette needs to be removed to create unmarked mutants (Bardarov et al. 2002).

Jain et al. (2014) developed an improved method for generating unmarked mutants in *Mtb* by combining specialized transduction with HR (Jain et al. 2014). In this study, the process for manipulating allelic exchange substrates (AES) for HR was optimized, including a hygromycin resistance cassette for selection, and a *sacB* gene for counterselection, both flanked by γδ‐resolvase recognition sites to facilitate marker excision. A shuttle phasmid, which is a conditionally replicating derivative of phage TM4, was developed with higher cloning and transduction capacities. The phage‐based transient γδ resolvase expression methodology developed allowed simple removal of the antibiotic marker (Jain et al. 2014). This system was successfully used to construct a drug‐sensitive double auxotroph *Mtb* mutant that, in vivo, showed lower virulence than the vaccine *M. bovis* BCG strain (Jain et al. 2014).

However, the early HR‐based allelic exchange techniques typically required a time‐consuming two‐step process involving the expression of an exogenous resolvase or recombinase from a replicative plasmid to allow the excision of the resistant marker, followed by the elimination of the replicative plasmid. An exception was the Xer‐method, which utilizes the endogenous XerC and XerD recombinases to resolve *dif* sites flanking a marker cassette, thereby enabling efficient gene disruption in *Mtb* (Cascioferro et al. 2010). For instance, in *Escherichia coli*, the XerC and XerD recombinases are essential for chromosome segregation during cell division, as they resolve chromosome dimers into monomers by recognizing the 28 bp of sequence present in the replication terminus region. Indeed, this site‐specific Xer recombination system is highly conserved among Gram‐negative and Gram‐positive bacteria (Cascioferro et al. 2010). This conservation underlines the evolutionary importance of maintaining genome stability by resolving chromosomal dimers during replication. In the context of *Mtb*, a slow‐growing bacterium, the presence of Xer‐like recombination machinery suggests a potential avenue for developing endogenous, site‐specific integration systems. This method was validated by generating untagged mutants in both *M. smegmatis* and *Mtb*. A deletion was introduced in the essential *dprE1* gene, by obtaining a merodiploid mutant, in which a second wild‐type copy of the gene was provided via a replicative plasmid, allowing targeted deletion without compromising cell viability (Cascioferro et al. 2010).

The construction of mutants by allelic exchange is further complicated by the relatively high frequency of illegitimate recombination in *Mtb* cells (Kalpana et al. 1991), which might be attributed to its slow growth rate. In contrast, the frequency of illegitimate recombination in fast‐growing mycobacteria such as *M. smegmatis* is low, as recombination occurs in a manner similar to that of *E. coli* and other bacteria (Kalpana et al. 1991). For this reason, the procedure by Cascioferro et al. (2010) was further improved to obtain multiple unmarked mutants of increased genetic stability by constructing variants of the natural *Mtb dif* site flanking a zeocin cassette. Exploiting this conserved recombination mechanism enabled more stable genomic insertions or deletions to be achieved, thereby improving the precision and efficiency of genetic manipulation in *Mtb*. The effectiveness of the system has been demonstrated by its ability to generate mutants in both the fast‐growing *M. smegmatis* and the pathogenic *Mtb*, highlighting its versatility across mycobacterial species (Boudehen et al. 2020).

The development and refinement of targeted gene disruption methods in *Mtb* have significantly advanced the genetic manipulation of this pathogen, enabling the generation of precise, unmarked mutants essential for functional genomics, vaccine development, and the study of mycobacterial pathogenesis.

### 2.1 Mutagenesis by Recombineering Method

A key limitation of the gene replacement strategies described until now is their requirement for large amounts of DNA (1–10 µg) while producing a low number of mutants. In contrast, site‐specific recombination is catalyzed by a recombinase and involves specific DNA sites, usually inverted repeats. Mycobacteriophages integrate their genome into *Mtb* chromosome through site‐specific recombination; this ability has been widely studied and adapted by researchers for targeted genetic manipulation in mycobacteria (Lee et al. 1991).

Given the inefficiency of HR in *Mtb*, to achieve higher recombination frequencies and to efficiently disrupt target genes, van Kessel and Hatfull (2007) developed the recombination‐based genetic engineering (recombineering) method. This inducible expression system employs the phage‐derived RecET (gp60/gp61) proteins from mycobacteriophage Che9c, whose constitutive expression can adversely affect bacterial growth.

A linear AES can be easily generated through plasmid digestion or overlapping PCR. Recombineering greatly increased the efficiency of allelic exchange compared to more traditional methods and has become the method of choice for this purpose.

A year later, the use of RecET to promote recombination by oligonucleotides in *Mtb* cells was better described (van Kessel et al. 2008). In this case, RecET can efficiently introduce a single nucleotide polymorphism (SNP) into the mycobacterial genome, which could be identified by PCR screening. This level of precision makes this technique particularly well suited to confirming the association of a selected mutation with drug resistance (Ioerger et al. 2013), or to transferring SNPs from clinical isolates to mycobacterial laboratory strains in order to test their relevance to a specific disease phenotype (Stucki and Gagneux 2013).

For example, using the recombineering method, Mori et al. (2020) demonstrated that Rv0579 is linked to resistance to TP53, a compound with antitubercular activity. Previously, *Mtb* spontaneous TP053‐resistant mutants were isolated, presenting the L240V mutation in Rv0579. By recombineering, new isogenic mutants harboring the same mutation in *Rv0579* were constructed and found to be resistant to TP053 (Mori et al. 2020).

Furthermore, Ioerger and collaborators (2013) isolated some *Mtb* mutants resistant to new antitubercular compounds harboring a mutation in the genes coding for possible drug targets. To confirm these findings, they constructed mutants having the same mutations and confirmed their drug resistance profile. So, interesting drug targets were found and confirmed, such as AspS, an aspartyl‐tRNA synthetase; Pks13, a polyketide synthetase involved in mycolic acid biosynthesis; MmpL3, a membrane transporter; and EccB3, a component of the ESX‐3 type VII secretion system (Ioerger et al. 2013). The generation of deletion mutants was significantly enhanced by combining the recombineering approach with specialized transduction, whereby phages carrying AES were directly transduced (Tufariello et al. 2014).

Finally, as previously illustrated with different examples, recombineering approaches are undoubtedly the most widely used method for knocking out non‐essential *Mtb* genes nowadays.

### 2.2 ORBIT: Oligonucleotide‐Mediated Recombineering Followed by Bxb1 Integrase Targeting

This method builds on the significant work conducted in G. Hatfull's laboratory on the Bxb1 phage integration system (Kim et al. 2003; Mediavilla et al. 2000; Keenholtz et al. 2016) and the Che9 RecET recombination system (Marinelli et al. 2012; van Kessel et al. 2008). These studies provided the fundamental basis for developing this procedure.

**Figure 2:**
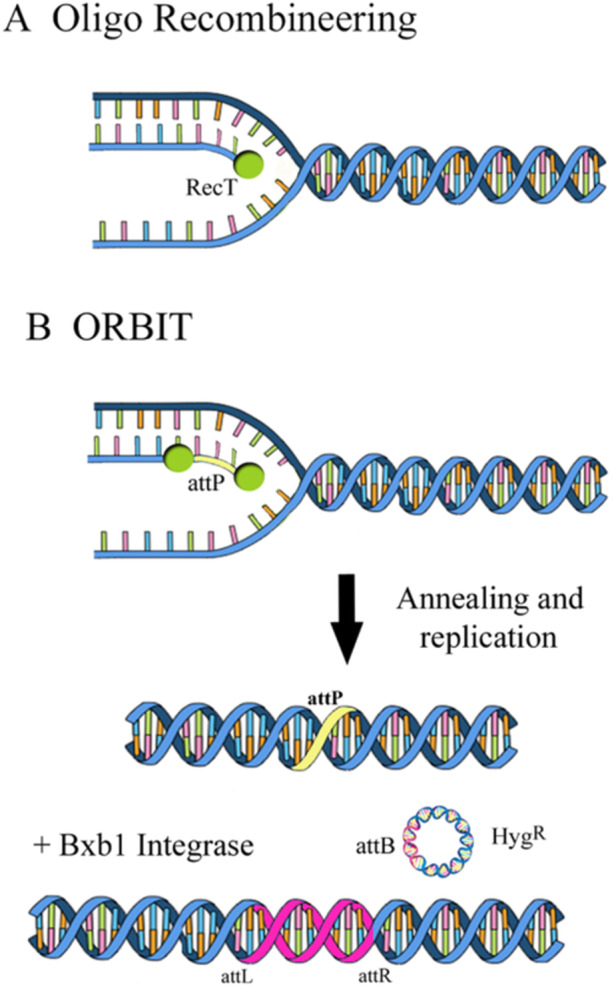
Example of oligo‐mediated recombination. In the first case (A), RecT binds to the electroporated oligonucleotide and transports it to the template lagging strand of the replication fork. Following annealing, the oligo is elongated by polymerase I and bound to neighboring Okazaki fragments. After the passage of the next replication fork, the chromosome containing a SNP is created. (B) In the ORBIT system, the oligonucleotide has an *attP* site of Bxb1, which is transported to the chromosome (as described above). Later, and at the same growth time, a co‐electroporated plasmid containing a non‐replicating *attB* site integrates into the chromosomal *attP* site. Depending on the target sequences present in the *attP*‐containing oligonucleotide and the payload sequences contained in the integrating plasmid, if present, deletions or fusions are generated (Murphy 2021).

Oligonucleotide‐mediated recombineering followed by the Bxb1 integrase targeting or ORBIT system utilizes the RecT protein and Bxb1 phage integrase to create gene deletions or insertions in *Mtb* genome (Figure 2) (Murphy et al. 2018).

To avoid the preparation of double‐stranded DNA recombination substrates, two efficient recombination systems were developed with the aim of producing an adaptable system for chromosome engineering. The Che9c annealase from the general HR system and the Bxb1 integrase from the site‐specific recombination system are combined in the ORBIT system (Murphy et al. 2018).

Through HR, which is facilitated by the phage Che9c RecT annealase, a synthetic “targeting oligonucleotide” is integrated into the chromosome. As this oligonucleotide contains a site‐specific recombination site for the directional Bxb1 integrase (Int), a “payload plasmid” with a cognate recombination site and a selectable marker can be simultaneously integrated. The payload plasmid acts as a modular delivery vector capable of carrying various genetic elements, such as selectable markers, epitope tags, fluorescent reporters, or regulatory sequences. This design allows for highly flexible genetic manipulation, eliminating the need for extensive cloning or multiple transformation steps. Drug‐resistant HRs can then be selected in a single step because of the target oligonucleotide and payload plasmid co‐transforming to generate a strain that expresses RecT and Int (Figure 2) (Murphy et al. 2018).

The ORBIT system has been employed for gene knockouts, reporter fusions, promoter replacements, and single‐base pair modifications in the chromosomes of both *Mtb* and *M. smegmatis* with remarkable efficiency. For knockouts, the oligonucleotide is then designed so that *attP* replaces the target gene. For C‐terminal tags, the oligonucleotide is tuned with the aim of inserting *attP* at the end of the coding sequence. In this way, the C‐terminal tag type is identified by selecting the plasmid to be co‐electroporated with the oligonucleotide from a library of pre‐existing payload plasmids. To validate the ORBIT system, the authors constructed several knock‐out mutants in both *Mtb* and *M. smegmatis* (Murphy et al. 2018).

Recently, the ORBIT system was employed to investigate the role of NucS endonuclease in the mismatch repair (MMR) of *M. smegmatis*. To this aim, the ORBIT system was extensively exploited to generate a *nucS* (*MSMEG_4923*) knock‐out mutant and a series of mutants harboring a defective hygromycin resistance gene with specific mismatches. Rivera‐Flores and coauthors demonstrated that NucS is capable of recognizing and repairing G‐G, G‐T, and T‐T mismatches in *M. smegmatis* cells, thereby establishing that both HR and non‐homologous end‐joining are not necessary for NucS‐promoted MMR, which is processed by a 5′‐3′ exonuclease (or 5′‐Flap endonuclease) (Rivera‐Flores et al. 2024).

Researchers have a very restricted toolkit with which to manipulate the genome of nontuberculous mycobacteria, which limits the possibility of studying emerging opportunistic pathogens such as *Mycobacterium abscessus* (*Mab*). It is interesting to note that the ORBIT system was successfully employed in *M. abscessus* (Cocorullo et al. 2025), for example, to study the mycothione reductase (Mtr) as a new possible drug target in *M. abscessus*. Indeed, mycothiol seems to be involved in protecting bacteria from reactive oxygen species. Interestingly, the *M. abscessus* ∆*mtr* mutant strain was generated by the ORBIT system; it was found to be more sensitive to bedaquiline in vitro and showed a decreased ability to grow inside macrophages (Piller et al. 2024).

These advanced recombineering systems achieve higher success rates, often increasing mutagenesis efficiency by more than an order of magnitude compared to classical HR, while reducing mutant construction time from several weeks to just a few days. These improvements not only streamline genetic manipulation in *Mtb* but also accelerate functional genomics studies, target validation, and, ultimately, the identification of new drug candidates (Murphy et al. 2015; Murphy et al. 2018). In addition, these methods can provide insights into mechanisms of action/resistance of drugs, such as activation or efflux (Ioerger et al. 2013; Wei et al. 2011).

## 3 Conditional Expression of Genes

Genes essential for mycobacterial growth have been widely studied due to their potential for drug development and their possible involvement in mycobacterial virulence (Degiacomi et al. 2023). However, the impossibility of eliminating these genes has made their investigation challenging, particularly in mycobacterial cells (Schnappinger et al. 2015). One possible strategy to overcome this issue is to place the transcription of the target gene under the control of an inducible promoter to modulate its expression. Indeed, inducible gene expression systems are a powerful tool for studying the function of a given gene and validating pharmacological targets in bacteria. The limited availability of expression vectors and the lack of well‐controlled promoters in mycobacteria have led to the search for regulated expression systems from other species that can be adapted for use in mycobacteria. This is due to the intrinsic challenges associated with mycobacterial gene regulation. Mycobacteria indeed possess unusual promoter architectures and complex transcriptional regulation, which is influenced by their high GC content and unique sigma factor repertoire. This often limits the predictability and tunability of gene expression. Furthermore, the slow growth rate of *Mtb* complicates the empirical optimization of promoter activity and induction kinetics. Endogenous regulatory systems are also constrained by metabolic and environmental responsiveness, which can interfere with experimental control. Consequently, heterologous inducible systems, such as tetracycline‐ or acetamide‐regulated promoters, have been explored to provide precise, temporally controlled, and reproducible gene expression; this facilitates functional studies of essential genes and enables the validation of novel drug targets.

In 1998, an expression vector harboring the highly inducible acetamidase promoter of *M. smegmatis* was constructed and used to induce high levels of production of an *M. leprae* antigen in *M. smegmatis* cells (Triccas et al. 1998). Acetamidase enables the microorganism to utilize specific amide compounds as a carbon source, and it is inducible (Parish et al. 1997; Triccas et al. 1998). Indeed, it is overexpressed by approximately 100‐fold in the presence of acetamide, compared to non‐inducible conditions. The biological system comprises five genes, including the *amiE* gene coding for acetamidase, and the regulation is mediated by the presence of two positive regulators (AmiC and AmiD) and one negative regulator (AmiA) (Roberts et al. 2003). However, the system presents limitations for its use in the development of conditional knockdowns, as complete repression of gene expression is not achievable, due to the presence of a very large region (the entire operator measures 1.4 kb) and an inducer (acetamide), which requires special growth media to ensure optimal activity. To overcome the limitations related to the plasmid size, a new expression vector was developed, in which the acetamidase regulon was minimized by deleting the *amiD* and *amiS* genes. This new version of the system has been optimized for producing mycobacterial recombinant proteins (Magaña Vergara et al. 2017). Furthermore, acetamide is not suitable for in vivo administration as it is considered a potential human carcinogen. Therefore, although the acetamidase expression system was initially effective in driving inducible gene expression in mycobacteria, it was subsequently abandoned in favor of more manageable alternatives due to several inherent limitations. First, high concentrations of acetamide were required for induction, which could interfere with cellular metabolism and impose metabolic stress. In addition, the system exhibited variable induction efficiency across different mycobacterial species and growth conditions, and suffered from leaky basal expression. This made the tight regulation of essential genes difficult.

Thus, over the past few years, several other genetically regulated expression systems have been developed for use in mycobacteria. It is important to note that these include inducible systems mediated by the repressor temperature‐sensitive TraR (Lim et al. 2000), nitrile (Pandey et al. 2009), and pristinamycin (Forti et al. 2009). In contrast to the acetamide system, newer systems, such as those induced by tetracycline or pristinamycin, offer greater tunability, reduced background expression, and simpler experimental control, making them more practical for functional genomics and target validation studies in *Mtb* (Forti et al. 2009; Evans and Mizrahi 2015).

### 3.1 TetR Systems

Currently, the most employed inducible systems rely on the use of tetracycline or anhydrotetracycline as a small inducer in concert with the Tet regulator. The TetR‐tetO system is one of the most efficient inducible systems, thanks to the possibility of obtaining a fine and tight regulation with a basal expression level. Interestingly, it is also used for gene regulation in eukaryotes. Due to their efficiency, tunability, and applicability in both in vitro and in vivo settings, the TetR‐regulated expression systems have become some of the most widely used tools in mycobacterial genetics. However, their significance is best demonstrated through specific experimental outcomes. For example, Tet‐inducible systems have been crucial for validating essential targets such as *InhA*, *RpoB*, and *KasA*, directly informing the development of frontline antitubercular drugs. Beyond target validation, the use of TetR‐based systems in murine infection models has demonstrated tight temporal control of gene expression, enabling the impact of essential gene depletion in vivo on bacterial survival to be assessed. These applications highlight the system's regulatory precision and translational relevance, bridging the gap between fundamental genetic studies and preclinical drug efficacy models. Together, these findings establish the TetR platform as a cornerstone of functional genomics and target‐based drug discovery in *Mtb* (Nagarajan and Sinha 2008; Carroll et al. 2005; Klotzsche et al. 2009).

The study of a biological mechanism provided the foundation for the development of Tet‐mediated inducible systems. Tetracyclines (tet) are a class of hydrophobic antibiotics that enter the cell by passive diffusion (Chopra and Roberts 2001). Gram‐negative bacteria can develop resistance mediated by TetR, a repressor regulating the expression of a family of proteins involved in the export of the antibiotic. In the absence of tetracycline, TetR associates with *tetO* palindromic sites in the operator, preventing both *tetA* and its own transcription. When tetracycline enters the cell, it tends to associate with bivalent ions, forming complexes. The binding of TetR to the complex results in a conformational change that causes the repressor to detach from the operators, allowing the transcription of *tetA* and of the repressor itself (Berens and Hillen 2003).

In 2005, three reports came out claiming these discoveries (Blokpoel 2005; Ehrt 2005; Carroll et al. 2005). Essentially, all three systems were based on the use of TetR and TetO regions inserted at the promoter level or within the intragenic region. TetR repressor derived from the Tn10 transposon of *E. coli* (Ehrt 2005; Carroll et al. 2005) or from an alternative tetracycline efflux system identified on a plasmid from *Corynebacterium glutamicum* (Blokpoel 2005). These systems are named collectively as TetON systems; in fact, the use of tetracycline as an inducer is a common feature across them. TetR protein works as an inducer, repressing the gene of interest. The addition of tetracycline or anhydrotetracycline is essential for the start of transcription. However, the presence of tetracycline can also pose a challenge, since removing the inducer to achieve gene silencing can be complex.

Therefore, an alternative model was proposed by Guo et al. (2007), developing a mutant (TetR r1.7) in which efficient repression of *lacZ* expression was achieved in *M. smegmatis* only in the presence of anhydrotetracycline (ATc). Using this strategy, they achieved silencing of the *secA* gene in a mutant (Guo et al. 2007). As the addition of ATc turns off transcription of the target gene, in this case, systems using reverse TetR are named “TetOFF.”

Another inducer that is worth mentioning is the pristinamycin (Forti et al. 2009), a naturally occurring antibiotic of the streptogramins group synthesized by *Streptomyces pristinaespiralis*, consisting of two components, pristinamycin IA and IB (Mast et al. 2011). The regulatory switch that confers resistance to *Streptomyces* producers consists of the pristinamycin resistance gene, *ptr*, encoding for an efflux pump, its promoter P_*ptr*_, and the transcription factor Pip activated with pristinamycin (Blanc et al. 1995). Pip is part of the TetR family of transcription factors. This system was the basis for the development of the inducible PipON system in mycobacteria, as P_*ptr*_ is a strong promoter, in both species *M. smegmatis* and *Mtb*, that can be efficiently repressed by Pip.

Later, the repressible promoter system TetR/PipOFF was developed for both fast‐ and slow‐growing mycobacteria to improve stringency and allow greater versatility in the construction of conditional knock‐down mutants (Boldrin et al. 2010). This system has been successfully used to repress target gene expression by up to 100‐fold upon induction, enabling the conditional knockdown of essential genes in *Mtb* (Ehrt 2005). Two chromosomally encoded repressors, TetR and Pip, are required to form the regulatory circuit, while the target gene must be placed under the control of P_*ptr*_ promoter, a strong promoter repressible by Pip. *TetR* is transcribed from a constitutive promoter and *pip* from a TetR‐dependent promoter. Therefore, in the absence of ATc, TetR binds to its two TetR operators (*tetO*) present in the TetR‐dependent promoter, switching off *pip* transcription and allowing expression of the gene of interest. In the presence of ATc, *pip* transcription is allowed due to inactivation of TetR, and, consequently, Pip represses the expression of the gene of interest. The TetR/PipOFF system offers robust and reversible control of gene expression, but its main disadvantage was the use of a strong promoter such as that from *S. pristinaespiralis* (P_*ptr*_). In fact, when the gene of interest is physiologically underexpressed, P_*ptr*_ causes overexpression and accumulation of the encoded protein. Thus, to obtain a phenotype and study the essentiality of the target gene, several culture passages were necessary to titrate down the protein product. Since it can be difficult to suppress genes physiologically expressed at low levels, the system was refined by mutagenizing the P_*ptr*_ promoter to generate promoters with varying degrees of transcriptional strength (Boldrin et al. 2018).

The techniques have been mostly applied to explore potential drug targets, generating conditional knockdown mutants for each gene involved in the studied biological pathway. A remarkable example was the application of TetR/PipOFF system to study the decaprenyl‐phospho‐D‐arabinofuranose pathway. The first‐line drug ethambutol and the benzothiazinones (Makarov et al. 2009) act on key enzymes involved in arabinan biosynthesis, which is essential for the mycobacterial cell wall. Several essential genes, such as *dprE1, dprE2, ubiA, prsA, rv2361c, tkt*, and *rpiB* were knocked down to identify additional potential targets in this pathway (Kolly et al. 2014). Another drug target, the CTP synthetase PyrG, was exploited to assess the potential of untargeted antimycobacterial compounds using a target‐based approach, involving the construction of conditional mutants with the PIP‐ON system (Mori et al. 2015; Esposito et al. 2017).

Finally, conditional mutants obtained in vitro using the Pip‐ON and the Pip‐OFF systems were characterized to confirm the essentiality and druggability of *canB*, encoding one of the three *Mtb* β‐carbonic anhydrases. This application also led to the identification of novel inhibitors (Degiacomi et al. 2023).

### 3.2 CRISPR‐Cas‐Based Strategies

Expanding the horizon of genome editing in mycobacteria, the discovery of the Clustered Regularly Interspaced Short Palindromic Repeats (CRISPR)‐Cas mediated technologies greatly facilitated genetic modifications in mycobacteria. There are several CRISPR systems for different organisms; one of the simplest is the CRISPR system from *Streptococcus pyogenes* (Qi et al. 2021). Over the last decade, they have rapidly evolved into a powerful genetic manipulation tool for mycobacteria (Choudhary et al. 2015; Singh et al. 2016; J. M. Rock et al. 2017; J. Rock 2019).

The CRISPR locus was initially studied as a phylogenetic marker due to its highly conserved nature (Kushwaha et al. 2020); consequently, little attention was given to its functional role. Indeed, since their discovery, almost 40 years ago (Ishino et al. 1987), CRISPRs have been considered a distinctive feature of bacterial and archaeal genomes. Nevertheless, only in the mid‐2000s was their function understood, consisting of a natural defense system against phage invaders (Mojica et al. 2005; Pourcel et al. 2005; Bolotin et al. 2005). Recent studies reveal that *Mtb* harbors a partially functional Type III CRISPR system that provides defense against foreign genetic elements through the ancillary RNase Csm6 (Grüschow et al. 2019). CRISPR‐associated proteins have emerged as a simpler tool for precise gene editing, facilitating the exploration of *Mtb* biology and its interactions with the host immune system (Ma et al. 2014), and for subsequent applications including whole genome sequencing, transcriptomic analysis, and metabolomic analysis.

The CRISPR–Cas mechanism is based on the following three functional stages: spacer acquisition, CRISPR RNA (crRNA) biogenesis, and interference (van der Oost et al. 2009).

During the first phase, also called adaptation, foreign DNA is detected, developed, and integrated into the CRISPR locus as a novel spacer. The second phase of crRNA expression allows transcription of the CRISPR locus, most often as a single pre‐crRNA, and its consecutive transformation into mature crRNAs that each include a single spacer. In the final interference step, the crRNA is used by an effector complex for the identification and destruction of any phage or plasmid that exhibits sequence complementarity with the crRNA spacer sequence. These three steps are especially performed by CRISPR‐associated proteins (Cas proteins), encoded by *cas* genes that flank CRISPR arrays (Wright et al. 2016). The CRISPR‐Cas basically uses small RNAs to orient and cut foreign nucleic acids distinctly by their sequence (Wright et al. 2016; Gasiunas et al. 2012; Garneau et al. 2010; Jinek et al. 2012).

However, CRISPR interference (CRISPRi) is indeed a promising system for modulating gene expression. The CRISPRi system uses the *Streptococcus thermophilus* Cas9 enzyme, which presents better performance features (magnitude of target gene knock‐down and reduced toxicity) in *M. smegmatis* (J. M. Rock et al. 2017). In fact, J. M. Rock et al. (2017) among eleven Cas9 orthologs identified the enzyme from S. thermophilus (dCas9Sth1) as the most effective for targeting gene deletions in mycobacteria, enabling robust gene knockdown.

The CRISPRi system has several advantages over classical gene mutagenesis methods in mycobacteria. It is the simplest and fastest method of programmable gene regulation. With this single plasmid platform, gene knockdown requires only the cloning of a unique targeting region of about 20 bp into the single guide RNA (sgRNA) scaffold. Furthermore, the CRISPRi system is inducible, allowing easier manipulation of essential genes. Finally, in CRISPRi systems, the extent of gene knockdown can be finely tuned through several experimentally controllable parameters, allowing precise modulation of target gene expression. One key determinant is the strength of the protospacer adjacent motif (PAM), which influences Cas9 enzyme binding affinity. Weaker PAM sequences result in reduced repression efficiency, providing a means to modulate the transcription of the target gene.

In addition, inducer concentration (typically ATc or doxycycline in Tet‐regulated dCas9 systems) offers dynamic, dose‐dependent control over dCas9 expression levels; lower inducer concentrations lead to partial repression, while higher levels achieve near‐complete silencing.

**Figure 3:**
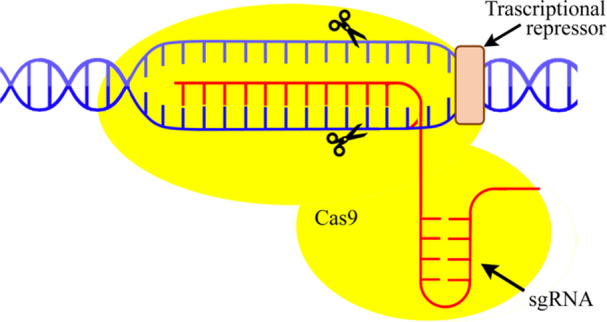
CRISPR interference (CRISPRi) system. It is an RNA‐based method for highly specific silencing of transcription in prokaryotic or eukaryotic cells.

A further level of tuning can be achieved by sgRNA truncation, where shortening the guide RNA weakens the RNA–DNA interaction, thereby decreasing binding stability and repression strength. Together, these strategies enable the generation of knockdown phenotypes with different levels of repression, an important feature for studying essential genes in *Mtb*, and contribute to the validity and reproducibility of CRISPRi‐based functional studies (Peters et al. 2016; Qi et al. 2021) (Figure 3).

In Rahman and colleagues (2022) reprogrammed an endogenous *M. tuberculosis* type III‐A CRISPR system to perform diverse genetic manipulations, including gene knock‐in/knock‐out, gene silencing, and whole‐genome RNA interference (RNAi) screening. The system operates through a two‐step cloning strategy in which a plasmid expressing a mini‐CRISPR array carrying a sgRNA is transformed into *Mtb* cells. To inhibit target gene transcription, this mini‐CRISPR array redirects the native CRISPR‐Cas (Csm) complex to the gene or transcript of interest, eliminating the need to introduce or express an exogenous *cas9* gene. As a result, this approach exhibits minimal cytotoxicity compared to Cas9‐dependent systems and functions without a PAM sequence requirement, broadening its targeting range. Furthermore, because multiple sgRNAs can be encoded within a single mini‐CRISPR array, the system enables true multiplexing, allowing simultaneous repression of multiple genes in one construct. This feature is particularly valuable for *Mtb*, a slow‐growing pathogen where conventional, serial genetic manipulation is time‐prohibitive. By permitting parallel silencing of several genes, CRISPRi multiplexing significantly accelerates functional genomic studies by shortening experimental timelines and enhancing the feasibility of complex gene‐network analyses relevant to pathogenesis and drug resistance.

The utility of CRISPRi in functional studies has been recently demonstrated by several studies. MmpL3, a well‐known target of Mtb, was silenced by using CRISPRi as a proof of concept for fast validation of gene essentiality study (McNeil and Cook 2019). More recently, Stupar et al. (2024), who characterized the two‐component regulatory system TcrXY, critical for *Mtb* in vivo survival. Using CRISPRi‐mediated silencing of *tcrX*, the gene encoding the TcrX response regulator, it was shown that this mutant exhibited reduced persistence during chronic mouse infection and increased susceptibility to first‐line anti‐TB drugs, rifampicin and isoniazid. These findings underscore the potential of optimized and multiplexed CRISPRi systems to accelerate the identification and validation of drug targets in *Mtb*.

## 4 Conclusion and Perspectives

The technical complexity and extended timelines associated with stable genetic manipulation in *Mtb* have long hindered comprehensive investigations into its pathogenic mechanisms and the validation of novel therapeutic targets (Bardarov et al. 2002; J. M. Rock et al. 2017). In particular, the generation of well‐regulated and reproducible conditional knockdown mutants remains a major challenge. Unlike fast‐growing model bacteria such as *E. coli* or *Bacillus subtilis*, for which mutant construction can often be completed within 2–3 days, the same process in *Mtb* may require 6–10 weeks, largely due to its ~24‐h doubling time and the need for prolonged selection and verification. Transformation efficiencies in *Mtb* are typically 10³–10⁴ CFU μg⁻¹ DNA, which is several orders of magnitude lower than in *E. coli*, severely limiting the recovery of recombinant clones. These difficulties are exacerbated by intrinsic physiological barriers, including an impermeable, lipid‐rich cell wall and resistance to standard selection antibiotics used as markers, which together limit the success rate of genetic manipulation (Armianinova et al. 2022; Li et al. 2022). The emergence of advanced genome editing technologies, particularly the CRISPR–Cas system, offers powerful strategies to overcome these limitations. CRISPR‐based tools allow precise, tunable, and high‐efficiency control of gene expression, enabling the functional dissection of essential genes even in slow‐growing mycobacteria (Choudhary et al. 2015; Singh et al. 2016). By substantially reducing the time required to generate targeted mutants and increasing reproducibility, these innovations are set to accelerate drug target validation, enable high‐throughput functional genomics, and deepen our understanding of *Mtb* physiology and virulence.

In conclusion, integrating CRISPR‐based technologies into *Mtb* research represents a transformative advancement that overcomes longstanding technical barriers and enables unprecedented precision in genetic manipulation. Unlike earlier inducible systems such as TetR, which relied on transcriptional control and often suffered from leaky expression or limited dynamic range, CRISPR‐based tools modulate gene activity directly at the DNA or RNA level through sequence‐specific sgRNAs. This level of precision allows for the fine investigation of essential gene function, complex pathogen–host interactions, and drug resistance mechanisms with a degree of control that was previously unattainable. Continued optimization and widespread application of these systems will be pivotal for accelerating functional genomics and developing next‐generation anti‐tubercular strategies.

## Author Contributions

**Alessandro Stamilla, Deborah Recchia, Laurent Roberto Chiarelli:** writing – review and editing, visualization. **Giovanni Stelitano, Maria Concetta Marturano, Edda De Rossi, Ludovica Maci:** visualization, methodology. **Maria Rosalia Pasca:** conceptualization, funding acquisition, writing – review and editing, project administration, supervision. **Giulia Degiacomi:** writing – original draft, investigation, conceptualization, visualization, methodology, funding acquisition.

## Ethics Statement

The authors have nothing to report.

## Conflicts of Interest

The authors declare no conflicts of interest.

## Data Availability

The authors have nothing to report.
